# Implementation of knowledge-based palliative care in nursing homes and pre-post post evaluation by cross-over design: a study protocol

**DOI:** 10.1186/s12904-018-0308-2

**Published:** 2018-03-22

**Authors:** Gerd Ahlström, Per Nilsen, Eva Benzein, Lina Behm, Birgitta Wallerstedt, Magnus Persson, Anna Sandgren

**Affiliations:** 1 Department of Health Sciences, Faculty of Medicine, Lund University, PO Box 157, SE-221 00 Lund, Sweden; 2 Department of Medical and Health Sciences, Division of Community Medicine, Linköping University, SE-581 83 Linköping, Sweden; 3 Department of Health and Care Sciences, Linnaeus University, SE-392 81 Kalmar, Sweden

**Keywords:** Complex intervention, Cross-over design, Elderly care, Evaluation, Frail elderly, Implementation, Implementation theory, Palliative care, Quality improvement, Residential care home, Staff education

## Abstract

**Background:**

The demography of the world is changing as the population is ageing. Because of this change to a higher proportion of older people, the WHO has called for improved palliative care for older persons. A large number of all deaths in the industrialised world occur while older people are living in nursing homes and therefore a key question becomes how the principles of palliative care can be implemented in that context. The aims of this study are: a) to describe a model of an educational intervention with the goal of implementing knowledge-based palliative care in nursing homes, and b) to describe the design of the evaluation of the effectiveness regarding the implementation of knowledge-based palliative care.

**Methods/design:**

A complex intervention is evaluated by means of a cross-over design. An educational intervention concerning palliative care consisting of five seminars during 6 months for staff and managers has been developed and conducted in 20 nursing homes in two counties. Before the intervention started, the feasibility was tested in a pilot study conducted in nursing homes not included in the main study. The intervention is evaluated through a non-randomized experimental design with intervention and control groups and pre- and post-assessments. The evaluation includes older persons living in nursing homes, next-of-kin, staff and managers. Data collection consists of quantitative methods such as questionnaires and register data and qualitative methods in the form of individual interviews, focus-group interviews and participant observations.

**Discussion:**

The research will contribute to new knowledge about how to implement knowledge-based palliative care in a nursing home setting. A strength of this project is that the Medical Research Council framework of complex intervention is applied. The four recommended stages, *Development*, *Feasibility and piloting*, *Evaluation* and *Implementation,* are combined for the educational intervention, which functions as a strategy to achieve knowledge-based palliative care in the nursing homes. Implementation is always a question of change and a good theoretical understanding is needed for drawing valid conclusions about the causal mechanisms of change. The topic is highly relevant considering the world’s ageing population. The data collection is completed and the analysis is ongoing.

**Trial registration:**

NCT02708498.

## Background

Palliative care has traditionally been provided for persons dying from incurable cancer. In contrast, older people dying of multiple morbidities or merely of “old age” have received far less of this type of care. One reason for this imbalance might be that it is more difficult to identify when the final stages of life begin for older persons. This knowledge is largely lacking outside specialist palliative care units [1]. Demographic changes leading to increasing numbers of older people have prompted calls by the World Health Organisation (WHO) for initiatives to improve palliative care for older persons [2].

Older people clearly have special needs of palliative care as they have multiple medical problems and diseases. The added burden of symptoms may be much greater than a single disease [1] due to decreased physical resources because of old age [3]. Yet the research indicates that an advanced age involves increased risk of poor health and disabilities, and that the final stage of life includes unnecessary suffering and decreased quality of life [1]. A high proportion of all deaths among older people occur while older people are living in residential care homes or nursing homes [4]. A key question is therefore how the principles of palliative care can be implemented in nursing homes [5, 6].

The goal of palliative care is to reduce suffering and promote quality of life for patients with progressive, incurable illnesses or injuries. The care should be person-centred and address physical, psychosocial and existential needs as well as provide support to the next of kin. Involving the older persons and their next of kin in the care process is therefore something that must be planned at an early stage when palliative care needs are identified as it is often not feasible to do this when the older persons are in the last days of life [1]. Nursing homes strive to provide good care for older persons in their final stages of life. However, the principles of palliative care which have been developed within specialized palliative care and hospices are too often not put into practice as recommended [1].

There is limited research on how the principles of palliative care can be implemented in nursing homes with the intention of providing the best possible palliative care and at the same time involving the older persons and their next of kin in the care process [5, 6]. Professionals need to provide palliative care based on older persons’ equal right to receive this type of care based on needs [7] and must also consider the fact that the frail older person is often single or has similar-aged next of kin, who may have health-related problems of their own [8, 9]. Based on the WHO guidelines for Palliative Care [10], the Swedish National Board of Health and Welfare has formulated the goal that everyone should have access to knowledge-based palliative care when the need arises, regardless of diagnosis, care form, age or where in the country a person lives [8].

There is an urgent need to improve palliative care throughout the world [1]. Several studies have reported that healthcare professionals have insufficient knowledge, skills and training in symptom management and other aspects of palliative care [11–13]. One considerable barrier is the lack of education provided for professionals regarding assessment and management of physical symptoms as well as psychological, social and existential concerns [14]. This study protocol describes an ongoing project in which an educational intervention based on knowledge-based palliative care principles is carried out in nursing homes in Sweden.

## Methods

### The aims of the study

The aims are: a) to describe an educational intervention with the goal of achieving knowledge-based palliative care in nursing homes, and b) to describe the design of the evaluation of the effectiveness regarding the implementation of knowledge-based palliative care. The intervention and evaluation is performed through a cross-over design.

### Setting

The Swedish welfare system is a largely tax-funded public system that provides equal access for everyone to healthcare, elderly care and social service based on each person’s need of support. The system is characterized by shared responsibility between two main authorities, the county councils and the municipalities. The county councils provide primary and specialist healthcare, being responsible for the investigation leading to diagnosis, medical treatment and follow-up examination. The municipalities are responsible for healthcare and social services as well as providing assistance for older persons living at home or in a nursing home. Nursing homes are accommodations where the residents live in their small flats with their own lease contract and with care provided around the clock. The right to a flat at a nursing home is based on the older person’s need of everyday care assessed by social workers in the municipality. This typically happens when the older person is too ill and frail to be able to continue to live an independent life in their previous home.

**Fig. 1:**
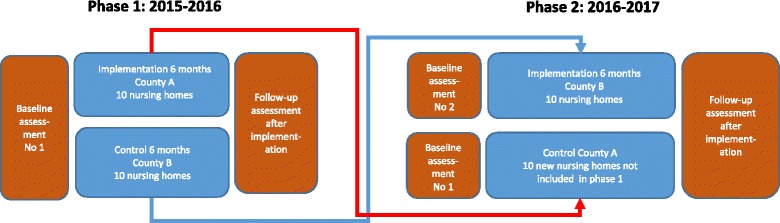
The cross-over design of the implementation strategy (educational intervention) and the experimental evaluation

The project *Implementation of knowledge-based palliative care* (KUPA-project) includes on the one hand 20 nursing homes in which the educational intervention was conducted over a period of 6 months in the form of seminars for managers and staff, on the other and 10 nursing homes which were only controls (Fig. 1). The intervention took place in two counties, Kronoberg and Skåne, in southern Sweden. The nursing homes in the project are a mix of larger (more than 100 older persons) and smaller (less than 25 older persons) nursing homes in the two counties, representing both urban and rural areas. The first contact with the nursing homes, for informing about the project and asking about the willingness to participate in the educational intervention and the evaluation, was facilitated through cooperation with the Skåne Association of Local Authorities and the Centre for Collaborative Palliative Care at Linnaeus University.

## Educational intervention

The purpose of the educational intervention is to implement knowledge-based palliative care in nursing homes. The intervention consists of a series of seminars that addresses the knowledge and skills deemed necessary to achieve knowledge-based palliative care in the nursing homes. Two Swedish documents have been used to identify key principles of palliative care: 1) a national care programme produced by the Regional Co-operative Cancer Centres [9] and 2) a national knowledge support document produced by the National Board of Health and Welfare [8]. Both documents are based on the WHO definition of palliative care [10] and the WHO reports [1, 2].

Before the start of this study the contents of the seminars and an educational booklet for use in the seminar series were designed after discussions with staff, informal carers and patients representing both hospital and community care. The educational booklet was based on published research on palliative care and the two knowledge documents [8, 9] and partly on a guide used in a previous research project in a city not included in the current project [15]. A pilot intervention was undertaken in the autumn of 2014, consisting of six 2–3 h seminars at 4 nursing homes using the educational booklet as study material for the participants. The feasibility of the pilot intervention resulted in a final version of the intervention which consisted of a series of five 2-h seminars about palliative care for staff. The seminars addressed the knowledge and skills that should be translated into routine practice in the nursing homes (Table 1). The seminars and the accompanying educational booklet addressed five themes: (1) the palliative approach and dignified care; (2) next of kin; (3) existence and dying; (4) symptom relief; and (5) collaborative care. The themes were adjusted somewhat based on the expressed needs and interests at each nursing home. The booklet also included recommended assignments to do as preparation before each seminar as well as assignments to complete after each seminar. The assignments have the effect that the staff take inspiration and “cases” from their daily work and bring the discussions from the seminars back to colleagues outside the seminar at their workplace in order to initiate work with healthcare improvements. A list of references for further self-studying by the staff was also given in the booklet [16].

**Table 1:** The content of the five seminaries in each of the 20 nursing homes

	Theme	Content
Seminar 1	The palliative approach and dignified care	The purpose of this theme is to increase the knowledge and understanding of the values on which palliative care is based, its ethics and manner of encountering the patient.
Seminar 2	Next of kin	The purpose of this theme is to increase knowledge and understanding of the situation and role of the next of kin and to consider how their need of support can better be met.
Seminar 3	Existence and dying	The purpose of this theme is to increase awareness and understanding of own thoughts concerning existence and death with dignity, this in order for the staff to feel secure in difficult conversations with patients and next of kin.
Seminar 4	Symptom relief	The purpose of this theme is to increase understanding of the importance of good alleviation of symptoms in palliative care and to promote the use of validated rating instruments.
Seminar 5	Collaborative care	The purpose of this theme is to bring about an increased understanding of the importance of teamwork, with particular focus on care collaboration between staff, patient and next of kin.

Each seminar group consisted of 8–10 participants and met every 4–5 weeks over a period of six months in each nursing home. The participants belonged to different professions and had different positions in the nursing homes: unit manager, registered nurses, assistant nurses, and other staff such as occupational therapists, physiotherapists and physicians. The seminar groups were led by two experienced clinical nurses and researchers from the field of palliative care and geriatric care (the authors LB and BW). The participants in the seminar groups reflected together on the contents of the booklet and discussed how they could apply the described knowledge and skills in their own everyday work to achieve knowledge-based palliative care.

The educational intervention targeted one group of staff members at each of the total 20 nursing homes. The staff were selected by the manager of each nursing home, with the instruction from the researchers (GA, LB and BW) to include a wide selection of professions. Approximately 200 staff members (assistant nurses, nurses, occupational therapists and physiotherapists) and 20 nursing home managers participated in the educational intervention. The educational intervention was conducted in the same way in the two counties and was completed in December 2016. The intention after the educational intervention was that the managers should ask staff members who participated in the intervention if they were willing and able to continue as seminar leaders and continue to educate the entire staff at each nursing home.

## Research design

The evaluation of the educational intervention had a non-randomized experimental design with 20 intervention and 20 control nursing homes (Fig. 1). The first 10 nursing homes which received the intervention at the beginning of the project were replaced by 10 new control nursing homes (that received the intervention after the project) as part of the cross-over design.

The evaluation of the educational intervention was carried out using a two-armed cross-over design in two counties (Kronoberg [County A] and Skåne [County B]) in southern Sweden. The experimental evaluation was conducted before and after the intervention was carried out. The cross-over design in this study is a way to reduce the risk of bias when not using randomization, to increase the control of differences in groups due to geographical area and culture, with increased validity of the results. The cross-over design also increases the number of nursing homes that receive the implementation.

**Fig. 2:**
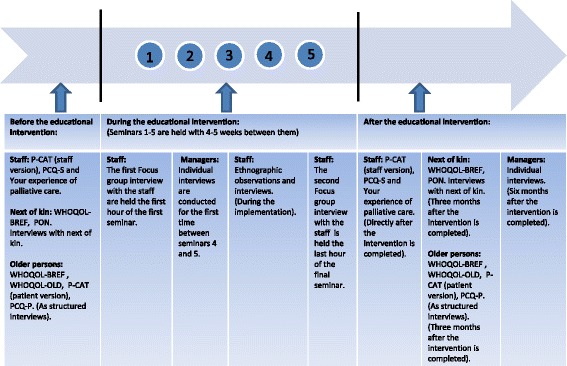
Flow chart of data collection in relation to the educational intervention

*Phase 1* (first arm) of the cross-over evaluation during 2015–2016 (the total time period was one year) included 10 intervention nursing homes in County A and a control group of 10 nursing homes in County B, as shown in Fig. 1. In *phase 2* (second arm) of the cross-over evaluation during 2016–2017 (the total time period was one year), the 10 control nursing homes in County B in the phase 1 were chosen to receive the intervention and 10 new nursing homes in County A, which had not received the intervention, were chosen as the control group. Thus a total of 30 nursing homes were included in the evaluation. The experimental evaluation with cross-over design was conducted in the same way in the two counties through validated measurement before (baseline) and after the intervention, as seen in Fig. 2.

### Framework for evaluation of the implementation of knowledge-based palliative care

In order to study the implementation of knowledge-based palliative care as a result of the educational intervention, the project applies a framework that identifies a number of determinants (acting as barriers and/or facilitators) which can affect the implementation (Table 2). Research in implementation science has established that a successful implementation depends on the interplay between: (1) effectiveness of the implementation strategies (e.g. in this project an educational intervention for care staff and managers) chosen to support the implementation; (2) characteristics of the “implementation object” (i.e. knowledge and skills concerning palliative care); (3) characteristics of the implementers (i.e. staff members’ attitudes, beliefs, motivation, etc.); (4) the target population (i.e. older persons at nursing homes and their next of kin); and (5) the context of the implementation [17–19]. The five interdependent determinants and how they are applied in this project are shown in Table 2.

**Table 2:** Implementation determinants in the evaluation

Determinant	Explanation	Applied and studied in this project
Characteristics of the implementation object	Features of the practices (program, service, methods, etc.) that are being implemented, e.g. knowledge-based or evidence-based practices that have been found to be effective in research.	In this project, the implementation object of palliative care is the palliative care practices described in the national knowledge-based documents produced by the National Board of Health and Welfare and the Regional Co-operative Cancer Centers.
Characteristics of the target population	Features of the receivers of the implemented practice, i.e. the ultimate target population, usually patients.	Investigate the characteristics of the target populations who are the frail older persons in nursing homes and their next of kin.
Characteristics of the implementers	Features of the professionals who use and deliver the practice. These “implementers” are those responsible for delivering the practice.	Investigate the characteristics of the implementers, who are all the nursing home staff as well as the managers working at the included nursing homes.
Effectiveness of implementation strategies used	Strategies chosen to facilitate the implementation of the practices. Implementation strategies (also referred to as implementation interventions) are conscious efforts to influence the implementation process in order to achieve desired practice changes.	Investigate the effectiveness of the educational intervention in Kronoberg County in 2015 and Skåne County in 2016. A comparison of data from the Swedish Palliative register from two years before and two years after the implementation will also be conducted to analyse the effectiveness of the implementation.
Characteristics of the context in which the implementation occurs	The context is the social environment in which implementation takes place. The context represents influences on the implementation process and impact that is, at least partially, beyond the control of the implementers and target population.	Investigate the characteristics of the context in nursing homes, in which implementation of knowledge-based palliative care takes place.

### Sampling and study participants

The selection procedure was initiated through including the nursing homes in the two counties (clusters) based on informed consent from leaders. Ten leaders in each county wanted to implement palliative care in the nursing home they were responsible for, and they were aware of their own role as a leader in this implementation. In the next step, the selection of number of participants in each county was based on the criteria for mixed method, i.e. for qualitative method and quantitative method respectively [20] (Table 3).

**Table 3:** Data collection in Kronoberg County and Skåne County during 2015 and 2017 before and after implementation of knowledge based palliative care as a cross-over design

Subjects	Method	Selection in the implementation nursing homes	Selection in the control nursing homes
Sub study 1: Older persons	Structured interview (based on WHOQOL-BREF (26 items), WHOQOL-OLD (24 items), P-CAT (13 items) and PCQ-P (17 items)	40 older persons before and three months after finished implementation (i.e. 9-month interval)	Same number and interval as in the implementation nursing homes
Sub study 2: The Swedish Register of Palliative Care (SRPC)	Anonymous data for deceased persons from the participating nursing homes and nationally from the Swedish Register of Palliative Care (SRPC)	7 selected questions from the Swedish Register of Palliative Care (SRPC)	Same as in the implementation nursing homes
Sub study 3: Next of kin	Qualitative interviews about quality of life and participation and two questionnaire, PON (32/36 items) and WHOQOL-BREF (26 items)	40 next of kin before and three months after finished implementation (i.e. 9-month interval)	Same number and interval as in the implementation nursing homes
Sub study 4: Next of kin	Two questionnaires, PON (32/36 items) and WHOQOL-BREF (26 items)	200 next of kin before and three months after finished implementation (i.e. 9-month interval)	Same number and interval as in the implementation nursing homes
	Psychometric study; test-retest, explorative factor analysis	See above	See above
Sub study 5: Staff	Focus group interviews (preparedness for implementation)	20 staff divided into 3 groups before and after the implementation is finished	Not done in the control nursing homes
Sub study 6: Staff	Questionnaires at two occasions: Your experience of palliative care (46 items), P-CAT (13 items) and the PCQ-S (14 items)	350 staff before and three months after finished implementation (i.e. 6-month interval)	Same number and interval as in the implementation nursing homes
Sub study 7: Managers	Two individual interviews with managers (organic preparedness for implementation)	20 managers at different levels-, during the implementation and 6 months after finished implementation (i.e. 9-month interval)	Not done in the control nursing homes
Sub study 8: Documentation	Diaries from the seminar group leaders, the participants evaluations of the seminars, documentation from board meetings as well as from the reference group meetings	All the available documentation between 2015 and 2017	
Sub study 9: Implementation context	An ethnographic study: Observations and qualitative interviews	Observations on 4 occasions at 5 nursing homes with a strategic selection of qualitative interviews during the implementation	Not done in the control nursing homes

The evaluation included both older persons and next of kin as the target groups that would benefit from a successful implementation as well as staff and managers who were the target groups performing implementation. The participants in the evaluation were recruited in equal numbers from both the intervention and control nursing homes. The persons were selected consecutively until the stipulated figures for each planned sub-study were reached (see Table 3).

- *Older persons*: The inclusion criteria for the older persons were ≥ 65 years without dementia and with enough energy to manage a structured interview during up to one hour.
- *The next of kin*: The inclusion criterion was someone who had a relation to one of the older persons living at the participating nursing homes but not necessarily family members or relatives.
- *Staff*: The inclusion criterion was staff (commonly assistant nurses, nurses, occupational therapists and physiotherapists).
- *Managers*: Managers at different levels with responsibility for the care and social service at the included nursing homes.
- More details about the characteristics of participants will be published in future studies (Table 3).

### Evaluation methods and measurements for older persons, next of kin, staff and managers

For the purpose of investigating the effectiveness of the implementation of knowledge-based palliative care through educational seminars, this evaluation study includes various methodological approaches, including qualitative and quantitative methods. The quantitative methods consist of questionnaires and registry data. The qualitative data consists of focus group interviews, individual interviews and participant observations.

#### Questionnaires

The following questionnaires were used in the intervention and control nursing homes to evaluate the intervention through before and after assessments (Table 3 and Fig. 2):

- *Quality of life*: The WHOQOL-BREF questionnaire (26 items) [21] and the WHOQOL-OLD questionnaire (24 items) [22] were used to assess quality of life for the older persons. The older persons answered the questions in structured interviews [22]. The next of kin answered the WHOQOL-BREF questionnaire [21] as a self-reported questionnaire.
- *The Person-centered Care Assessment Tool (P-CAT)* (13 items) was used to measure the extent to which an elderly care setting was person-centred [23, 24]. Person-centred care is increasingly regarded as being synonymous with best quality care. In this project, both the patient version and the staff version were used.
- *The Person-Centered Climate Questionnaire (PCQ)* exists both in a patient (PCQ-P) (17 items) and a staff version (PCQ-S) (14 items). The instrument was used to assess to what extent the climate of care environments was person-centred [25, 26].
- *The participation of next of kin in care in nursing homes* (PON) is a questionnaire about palliative care at nursing homes, which consists of 32 or 36 items. We found that there was a lack of instruments designed to measure the participation of next of kin in palliative care in nursing homes. In order to deal with this, a questionnaire regarding the next of kin’s participation was developed before the beginning of the project. A literature review of previous research about the next of kin’s participation was performed, including all types of care in general but with particular focus on palliative care and the care at nursing homes. The systematic literature review resulted in a questionnaire with 32 items about next of kin’s participation in palliative care. Since death among the older persons at nursing homes is a fact to be reckoned with, it became necessary to construct a slightly altered version with 36 items for use in case the older person had died between our data collection points. A psychometric evaluation of the questionnaire is planned.
- *Your experience of palliative care*: The staff also answered a questionnaire with 46 items, newly constructed to capture changes in health professionals’ attitudes, knowledge and assessment of the quality of palliative care; one month before and within one month after finishing the implementation intervention. Psychometric testing of the questionnaire is in process (Årestedt et al. not published).

#### Registry data

Data from the *The Swedish Register of Palliative Care (SRPC)*, [27] were extrapolated and used both in a descriptive part (to describe the condition before and after the intervention) as well as an analytic part. The SPRC consists of 27 questions relating to, among other things, management of symptoms, oral health, presence of pressure ulcers, if next of kin were offered a bereavement follow-up and if anyone was present at the moment of death.

#### Interviews and participant observations

*The interviews with the next of kin* followed a semi-structured interview guide including two theme areas about quality of life and participation. The interviews began with open-ended questions about quality of life and then about participation. The number and formulation of follow-up questions within each theme area depended on the richness of the participant’s answer to the open-ended question.

*Focus group interviews with the staff and unit managers* participating in the educational intervention at the nursing homes were chosen to investigate the staff’s preparedness before the intervention and their experiences of the implementation process of palliative care in elderly care after the intervention. Focus group interviews emphasise the interaction between respondents with a common frame of reference [28]. The focus groups consisted of 5–12 persons as well as a moderator who led the discussion and an observer researcher. Immediately after each interview, the observer made field notes, including a summary of the areas discussed, the order of speakers and the group dynamics, to ensure the quality of each group session [29].

*Individual interviews with managers* about the organizational readiness to change [30] in order to achieve knowledge-based palliative care were conducted through face-to-face interviews using a semi-structured interview guide. The interviews were conducted on two occasions, both during and after the educational intervention. The interview during the intervention focused on the readiness of their organisation and management level to implement palliative care and the interview after the intervention focused on whether implementation was ongoing or planned. To obtain comprehensive data, we attempted to create a dialogue designed to capture the participant’s experience of the phenomena of interest [31].

*Participant observations* were used as a method to explore the implementation context at the nursing homes. The method was chosen to obtain a deeper understanding of how palliative care emerged in everyday work. Following staff members in the nursing home setting makes it possible to study their experiences. This form of observation is called *go-along* [32], which is a concept that emphasises the perceptions of place and space to explore the individual’s lived experience. Informal conversations that occur between the staff and the researcher during the observation are also important [33]. Participant observations also involve studying the special material to be handled, such as medications and assistive devices, but also everyday objects in the nursing home. The social relations between staff and the older person are in focus: how such relations are expressed, negotiated and transformed, and how meaning is created.

### Data analysis

Both quantitative and qualitative analysis methods will be used. The quantitative evaluation through questionnaires are related to the goals of palliative care: a) the older persons’ quality of life (investigated with WHOQOL-OLD and WHOQOL-BREF) and experiences of person-centred care (P-CAT) and climate (PCQ-P*)*, b) the next of kin’s quality of life (WHO-BREF) and participation in the care process (PON) and c) the staff’s experiences of palliative care (Your experience of palliative care) and person-centred care (P-CAT) and climate (PCQ-S). Besides being used to generate descriptive statistics, the quantitative data of the questionnaires and registry data (SRPC) will be analysed using methods applicable to between-group (intervention group compared with control group) and within-group comparisons (after data compared with before data in the intervention group and the control group, respectively).

The selection of statistical methods aside from descriptive statistics in the study in question will be based on whether the data are distributed normally, the scale level of the data, recommendation in the manuals for WHOQOL-BREF [21] and WHOQOL-OLD [22] and the assessment of the current statistical power. Specifically, a Wilcoxon rank sum test will be used to compare data across independent groups, and a Wilcoxon signed rank test will be used to compare paired data as between after and before. For independent samples, the Mann-Whitney *U* test will be applied; or for categorical data, either the Pearson chi-square test or Fisher’s exact test (if any expected cell value turns out to be less than 5). To examine whether there are differences between urban and rural areas chi-2 tests will be used. Analyses will be performed using IBM SPSS Statistics version 23. A two-tailed *p* value of < 0.05 will be regarded as statistically significant.

The qualitative part of the evaluation is also related to the goals of palliative care, including the next of kin’s quality of life and participation in the care process. Implementation of key principles is followed through staff’s and managers’ experiences of organizational readiness to change [30], contextual influences and barriers and facilitators of implementing knowledge-based palliative care in the nursing homes. Qualitative data from the focus group interviews [28, 29], individual interviews [31] and field notes from participant observations [32, 33] will be analysed with different qualitative content analysis methods. All interviews and field notes are transcribed verbatim. The interviews conducted before the implementation intervention will be coded through an inductive process in the sense that themes will emerge through comparisons of parts and wholeness within an interview and between interviews [34, 35]. Interviews performed after the intervention will be coded through a mainly deductive qualitative analytical process [35, 36] based on the findings before the implementation. The process of qualitative content analysis will be supported by the Nvivo software version 11. The field notes and the individual interviews in connection with the participant observations will be analysed with ethnographic methods [32, 33]. This means that an inductive thematic approach will be applied in the qualitative analytical process. Field notes from the observations and interviews will be taken as a whole and scrutinized repeatedly in order to highlight patterns within the categories and identify themes. The thematic analysis will then continue with highlighting and coding of the content into two categories: “talking” and “doing”.

## Discussion

This project is aimed at implementing knowledge-based palliative care in nursing homes by means of providing an educational intervention for managers and staff. The project will contribute scientific knowledge about the educational intervention which conveys key principles of knowledge-based palliative care and about how palliative care can be achieved in nursing homes, which have not traditionally focused on palliative care.

The project employs a cross-over design, with intervention and control nursing homes (in total 30 nursing homes), which is a strategy for handling the weaknesses that are embedded in complex interventions [19, 21] such as the educational intervention in this project. A complex intervention according to the Medical Research Council (MRC) [37] is defined as an intervention that features several interacting components, with a range of possible outcomes, or variability. This complexity threatens construct validity in that the effects are difficult to attribute to certain components or specific “active ingredients” of the intervention. The widely used MRC framework comprises four stages: Development, Feasibility/piloting, Evaluation, and Implementation [19, 21]. These stages involve different key functions and activities at each stage. Even though these are presented as stages, they often do not follow a linear or even a cyclical sequence [19–21].

The first stage of the MRC framework *Development*, applied to this project, consisted of a literature review of studies concerning palliative care for older people, implementation and implementation theory. The literature review identified two recently published national knowledge documents with state of the art palliative care knowledge [8, 9]. An educational intervention programme based on the literature review and the contents of the two knowledge documents was developed for staff and transformed into a booklet. The seminars were designed to use reflective practice activities [38] along with the booklet. To develop and conduct a complex intervention is not enough without evaluation for valid conclusions [37, 39]. Therefore the evaluation in this project addresses several different aspects, including the results of the educational intervention with regard to staff members, the older persons and their next of kin and various barriers and facilitating aspects of the implementation of knowledge-based palliative care in the nursing homes.

Research in implementation science has demonstrated the importance of planned strategies to achieve a more evidence-based healthcare. Without active facilitation of implementation, the pathway from research-based knowledge to routine practice tends to be lengthy and fraught with difficulties [40]. The educational intervention in this project is a concerted strategy to achieve state-of-the-art palliative care in nursing homes. Although the overall evidence of the effectiveness of educational interventions to change healthcare professionals’ behaviours is not overly strong, this was still deemed to be the most relevant, effective and feasible strategy to achieve the goals of attaining knowledge-based palliative care in the nursing homes. A Cochrane review of 81 studies comparing education meetings (encompassing seminars, workshops, lectures, courses) for healthcare professionals with no intervention found the effect was most likely to be small [41].

Recognizing the considerable challenge of changing professional practice by means of educational interventions, the seminars were specifically designed and executed to account for important findings from learning, organizational and implementation research. It was reasoned that learning based on formal education alone would not be enough to meet the challenges of providing knowledge-based palliative care of high quality in nursing homes. The emphasis on discussion in the seminars is based on an awareness that important learning results from reflecting on experiences [42]. Reflection is also facilitated by adapting some of the seminar contents to the specific needs and interests in the different nursing homes, thus making the seminars as relevant as possible to the participants. The concept of reflection is generally understood as a means of translating experience into learning, by examining one’s responses, beliefs, and actions, to draw conclusions to enable better choices or actions in the future [42–44].

The seminars seek to integrate research-based knowledge (previous research and the knowledge-based documents) and practice-based knowledge (based on the participants’ experiences) as the participants’ own experiences are discussed in relation to the various palliative care topics that are covered in the seminars. Deeper learning can be facilitated by purposively using research-based knowledge to challenge established ways of seeing and understanding the world [45, 46]. It has been argued that research-based knowledge makes it possible to move beyond the specific “here-and-now” circumstances of current work practices and to show new opportunities for acting [47, 48]. Studies on research use in healthcare have demonstrated that research can be used by practitioners to consider their work practices in a new light and reflect on the underlying taken-for-granted assumptions that shape their work.

Furthermore, the seminars were planned to encourage active engagement by the participants. Active learning is generally defined as any instructional method that engages participants in the learning process. This type of education is often contrasted with traditional lectures where the participants passively receive information from an instructor [49]. Research has shown that healthcare professionals’ practice change is more likely by means of interactive education than through using more passive lectures and similar formats [41].

Another key foundation of the educational intervention was to involve the managers at the nursing homes. This was a conscious strategy intended to facilitate development of a culture that was more conducive to palliative care. Managers who act as leaders have the potential to influence the culture by imposing their values, norms and assumptions on the staff, influencing staff members in respect of how to perceive, think, feel and behave based on their own convictions [50, 51]. Culture has been defined as the shared values (important and lasting ideals and preferences for certain behaviours), norms (beliefs about acceptable behaviours) and assumptions (unspoken beliefs and expectations) among members of a group [52].

The second stage of the MRC framework, *Feasibility and piloting*, included a pilot study which resulted in a reduction from six seminars to five. The third stage, *Evaluation*, and the fourth stage, *Implementation*, were combined in terms of the educational intervention, which functioned as an implementation strategy to achieve knowledge-based palliative care in the nursing homes.

### Implications

Transforming research-based knowledge into routine practice by means of educating the staff is necessary to realise the goals of knowledge-based palliative care. We believe that our project will contribute new knowledge about how to implement knowledge-based palliative care principles, based on the WHO definition of palliative care in a nursing home setting. The topic is highly relevant considering the world’s projected demographic development and will be of importance to policymakers and professional carers at nursing homes all over the world.

## References

[1] Davies E, Higginson IJ (2004). Better palliative care for older people.

[2] Hall S, Petkova H, Tsouros A, Constantini M, Higginson I (2011). Palliative care for older people: better practices.

[3] Bilotta C, Bowling A, Case A, Nicolini P, Mauri S, Castelli M, Vergani C (2010). Dimensions and correlates of quality of life according to frailty status: a cross-sectional study on community-dwelling older adults referred to an outpatient geriatric service in Italy. Health Qual Life Out.

[4] Broad JB, Gott M, Kim H, Boyd M, Chen H, Connolly MJ (2013). Where do people die? An international comparison of the percentage of deaths occurring in hospital and residential aged care settings in 45 populations, using published and available statistics. Int J Public Health.

[5] Beck I (2013). To focus on the “thing” in a world of doing: support for the staff in a palliative approach to care and care for the elderly. (in Swedish).

[6] Orrung Wallin A (2013). Job satisfaction, strain and stress of conscience among nurse assistants working in residential care for older people.

[7] 1982:763 Health and Medical Services Act (In Swedish: Hälso- och sjukvårdslag). In: Edited by MoHS A. Stockholm: Min. of Health & Social Affairs; 1982.

[8] National Board of Health and Welfare: The National Knowledge Support Document for good palliative Care at the end of life (in Swedish). In: Edited by (Socialstyrelsen) Eb. Stockholm: National Board of Health and Welfare; 2013.

[9] The National Care Program for Palliative Care 2012–2014 (In Swedish). Stockholm: Regional Co-operative Cancer Centers (Regionala cancercentrum i samverkan); 2012.

[10] Connor SR, Sepulveda Bermedo MC. Global atlas of palliative care at the end of life. In: World Palliative Care Alliance. London: WHO; 2014.

[11] Bruera E, Willey JS, Ewert-Flannagan PA, Cline MK, Kaur G, Shen L, Zhang T, Palmer JL (2005). Pain intensity assessment by bedside nurses and palliative care consultants: a retrospective study. Support Care Cancer.

[12] Linder JF, Blais J, Enders SR, Melberg SE, Meyers FJ (1999). Palliative education: a didactic and experiential approach to teaching end-of-life care. J Cancer Educ.

[13] Levine S, O’Mahony S, Baron A, Ansari A, Deamant C, Frader J, Leyva I, Marschke M, Preodor M (2017). Training the workforce: description of a longitudinal interdisciplinary education and mentoring program in palliative care. J Pain Symptom Manag.

[14] Wittenberg E, Ferrell B, Goldsmith J, Smith T, Ragan S, Glajchen M, Hanzo G. Textbook of palliative care communication. Oxford: Oxford University Press; 2015.

[15] Beck I, Jakobsson U, Edberg AK (2014). Applying a palliative care approach in residential care: effects on nurse assistants’ experiences of care provision and caring climate. Scand J Caring Sci.

[16] Behm L, Wallerstedt B, Persson M, Ahlström G (2017). Educational seminars about palliative care - a way to increase knowledge in nursing homes. (in Swedish).

[17] Greenhalgh T, Robert G, Macfarlane F, Bate P, Kyriakidou O (2004). Diffusion of innovations in service organizations: systematic review and recommendations. Milbank Q.

[18] Damschroder LJ, Aron DC, Keith RE, Kirsh SR, Alexander JA, Lowery JC (2009). Fostering implementation of health services research findings into practice: a consolidated framework for advancing implementation science. Implement Sci.

[19] Nilsen P (2015). Making sense of implementation theories, models and frameworks. Implement Sci.

[20] Richards DA, Hallberg IR (2015). Complex interventions in health: an overview of research methods.

[21] Harper A, editor. WHOQOL-BREF INTRODUCTION, ADMINISTRATION, SCORING AND GENERIC VERSION OF THE ASSESSMENT. Geneva: World Health Organization; 1996.

[22] Power M, Quinn K, Schmidt S, Group W-O (2005). Development of the WHOQOL-old module. Qual Life Res.

[23] Edvardsson D, Fetherstonhaugh D, Nay R, Gibson S (2010). Development and initial testing of the person-centered care assessment tool (P-CAT). Int Psychogeriatr.

[24] Sjogren K, Lindkvist M, Sandman PO, Zingmark K, Edvardsson D (2012). Psychometric evaluation of the Swedish version of the person-centered care assessment tool (P-CAT). Int Psychogeriatr.

[25] Edvardsson D, Sjogren K, Lindkvist M, Taylor M, Edvardsson K, Sandman PO (2015). Person-centred climate questionnaire (PCQ-S): establishing reliability and cut-off scores in residential aged care. J Nurs Manag.

[26] Bergland A, Hofoss D, Kirkevold M, Vassbo T, Edvardsson D (2015). Person-centred ward climate as experienced by mentally lucid residents in long-term care facilities. J Clin Nurs.

[27] Martinsson L, Heedman PA, Lundstrom S, Fransson G, Axelsson B (2011). Validation study of an end-of-life questionnaire from the Swedish register of palliative care. Acta Oncol.

[28] McLafferty I (2004). Focus group interviews as a data collecting strategy. J Adv Nurs.

[29] Krueger R, Casey MA (1994). Focus groups : a practical guide for applied research.

[30] Weiner BJ (2009). A theory of organizational readiness for change. Implement Sci.

[31] Kvale S, Brinkmann S (2009). InterViews: learning the craft of qualitative research interviewing.

[32] Kusenbach M (2003). Street phenomenology: the go-along as ethnographic research tool. Ethnography.

[33] Pink S (2009). Doing sensory ethnography.

[34] Graneheim UH, Lundman B (2004). Qualitative content analysis in nursing research: concepts, procedures and measures to achieve trustworthiness. Nurs Educ Today.

[35] Hsieh HF, Shannon SE (2005). Three approaches to qualitative content analysis. Qual Health Res.

[36] Elo S, Kyngas H (2008). The qualitative content analysis process. J Adv Nurs.

[37] Craig P, Dieppe P, Macintyre S, Michie S, Nazareth I, Petticrew M (2006). Developing and evaluating complex interventions: new guidance.

[38] Roessger KM (2013). Investigating the impact of formal reflective activities on skill adaptation in a work-related instrumental learning setting.

[39] Craig P, Dieppe P, Macintyre S, Michie S, Nazareth I, Petticrew M, Medical Research Council G (2008). Developing and evaluating complex interventions: the new Medical Research Council guidance. BMJ.

[40] Wallace J (2013). Lost in translation: transferring knowledge from research to clinical practice. Adv Psychiatr Treat.

[41] Forsetlund L, Bjorndal A, Rashidian A, Jamtvedt G, O'Brien MA, Wolf F, Davis D, Odgaard-Jensen J, Oxman AD (2009). Continuing education meetings and workshops: effects on professional practice and health care outcomes. Cochrane Database Syst Rev.

[42] Kolb DA. Experiential learning: experience as the source of learning and development. Englewood cliffs: Prentice-Hall, cop; 1984.

[43] Dewey J (1998). How we think: a restatement of the relation of reflective thinking to the educative process.

[44] Schön DA. The reflective practitioner: how professionals think in action. New York: Basic Books, cop; 1983.

[45] Nilsen P, Nordstrom G, Ellstrom PE (2012). Integrating research-based and practice-based knowledge through workplace reflection. J Work Learn.

[46] Dopson S, Fitzgerald L. Knowledge to action?: evidence-based health Care in Context. [Electronic resource]. Oxford: Oxford University Press; 2005.

[47] Svensson L: Interaktiv forskning - för utveckling av teori och praktik: Stockholm : Arbetslivsinstitutet, cop. 2002; (Stockholm: Elander Gotab); 2002.

[48] White S, Fook J, Gardner F (2006). Critical reflection in health and social care.

[49] Bonwell CC, Eison JA, Association for the Study of higher E, Eric clearinghouse on higher education WDC, George Washington Univ WDCSoE, human D: active learning: creating excitement in the classroom. 1991 ASHE-ERIC higher education reports; 1991.

[50] Gill R (2011). Theory and practice of leadership.

[51] Schein EH (2010). Organizational culture and leadership.

[52] Bang H, Johansson G. Organization culture. 2nd Ed. (in Swedish). Lund: Studentlitteratur; 1999.

